# Targeting dendritic cells in pancreatic ductal adenocarcinoma

**DOI:** 10.1186/s12935-018-0585-0

**Published:** 2018-06-18

**Authors:** Anton Deicher, Roland Andersson, Bobby Tingstedt, Gert Lindell, Monika Bauden, Daniel Ansari

**Affiliations:** 1 Department of Surgery, Clinical Sciences Lund, Lund University and Skåne University Hospital, 221 85 Lund, Sweden; 2 Faculty of Medicine, Ruprecht-Karls-Universität Heidelberg, Heidelberg, Germany

**Keywords:** Pancreatic cancer, Dendritic cells, Antigen presenting cell, Immunotherapy, Vaccine

## Abstract

Dendritic cells (DC) are an integral part of the tumor microenvironment. Pancreatic cancer is characterized by reduced number and function of DCs, which impacts antigen presentation and contributes to immune tolerance. Recent data suggest that exosomes can mediate communication between pancreatic cancer cells and DCs. Furthermore, levels of DCs may serve as prognostic factors. There is also growing evidence for the effectiveness of vaccination with DCs pulsed with tumor antigens to initiate adaptive cytolytic immune responses via T cells. Most experience with DC-based vaccination has been gathered for MUC1 and WT1 antigens, where clinical studies in advanced pancreatic cancer have provided encouraging results. In this review, we highlight the role of DC in the course, prognosis and treatment of pancreatic cancer.

## Background

Ductal adenocarcinoma of the pancreas has the highest mortality rate amongst all major human tumors [1]. Due to the insidious nature of the disease, approximately 85% of patients have advanced disease at presentation. Survival rates have remained stagnant in the last decades and there is a lack of early detection tools and effective treatment [2]. In Europe, the median survival for pancreatic cancer is 4.6 months and only 3% of patients survive beyond 5 years [3]. Metastatic pancreatic cancer confers an especially poor prognosis and treatment progress has been slow. Multiagent chemotherapy has been shown to prolong survival by a few weeks to a couple of months, but toxicity remains a challenge and it is still difficult to identify responders to treatment [4]. There is an urgent need to develop new types of therapy based on individual tumor genotype/phenotype in order to increase survival rates and reduce overtreatment.

Traditionally, immunotherapy has had little success in solid tumors. However, recent progress in immuno-oncology, especially in melanoma and renal cancer, has revitalized the concept of harnessing the patient’s own immune system against the tumor [5]. Recent molecular mapping has revealed an immunogenic subtype of pancreatic cancer that may be targetable through immune treatment [6]. Furthermore, extensive immunoprofiling has shown that long-term survivors of pancreatic cancer are characterized by improved neoantigen quality and enhanced immune response [7].

Dendritic cells (DC) are professional antigen-presenting cells at the intersection of innate and adaptive immunity, initiating, directing and modulating immune responses. DCs are described as a heterogeneous population to match a variety of microenvironmental conditions [8]. Here, we review the role of DCs in the development, progression and potential therapy of pancreatic cancer.

### Paucity of dendritic cells in pancreatic cancer

Immune escape is one of the hallmarks of cancer [9]. Antigen presentation by DCs is essential to effective antitumor T cell responses. DCs are rare in the pancreatic tumor microenvironment and the cells are located at the edges of the tumor [10]. Systemically, decreased levels of blood DCs have been demonstrated in patients with pancreatic cancer [11]. Of note, increased circulating levels of DCs have been associated with better survival in patients with pancreatic cancer [11–13]. Furthermore, surgical removal of the pancreatic tumor was shown to improve blood DC function, supporting a tumor-derived influence on immune function [14].

Chronic pancreatitis is a major risk factor for pancreatic cancer. However, the molecular mechanisms bridging these entities are not well understood. It has been postulated that chronic inflammation not only promotes tumor development through the release of e.g. growth factors, but also indirectly by impairing the ability of DCs to activate immune responses against the tumor [11].

### Mechanisms of dendritic cell suppression

The host immune reaction to pancreatic cancer is reported to change from immune surveillance to immune tolerance during disease progression. This is mediated by CXCL17 and ICAM2 [15]. Furthermore, tumor-derived cytokines, such as TGF-beta, IL-10, and IL-6, have been reported to suppress DC survival and proliferation [16]. Expansion of immature myeloid cells in the circulation as well as the spleen might further compromise the immune response. Levels of circulating myeloid derived suppressive cells (MDSCs) have been reported to be increased in pancreatic cancer, which may promote tumor progression [17, 18]. MDSCs produce nitric oxide (NO) and inhibit DC activation in pancreatic cancer [19].

### Subpopulations of dendritic cells

DC can differentiate into distinct subpopulations depending on microenvironmental stimuli, leading to proliferation of myeloid DCs that induce Th1 cell activation or plasmacytoid DCs that facilitate immunosuppressive T cell development. Tumor-derived cytokines have been reported to induce a tolerogenic plasmacytoid DC phenotype [16]. Furthermore, recent data suggest the existence of a specific subset CD11b^+^ DCs that establish an immunosuppressive microenvironment, which favors metastatic progression through the expansion of regulatory T cells (Tregs) and suppression of CD8+ T cells [20]. These findings indicate that pancreatic cancer is characterized not only by reduced numbers of DCs, but also a complex modulation of DC subpopulations, which affects tumor development.

### Maturation of dendritic cells

Defective maturation of DCs may contribute to the development of a tumor-tolerant immune status. It has been found that semi mature blood DCs exist in pancreatic cancer patients, likely mediated by inflammatory factors released from the tumor [21]. These DCs have lost their ability to act as professional antigen presenting cells and activate T cell responses, and this leads to suppression of adaptive immune responses.

### Aberrant antigen presentation

Altered antigen processing and presentation is an important mechanism by which cancers evade the immune system. Pancreatic tumors are characterized by a loss or down-regulation of antigen-processing and antigen presenting molecules, including the human leucocyte antigen (HLA) class I and transporter for antigen presentation (TAP). Reduction of HLA class I expression was observed in 76% of pancreatic cancer cases and 53% of cases had reduced TAP expression [22].

### Crosstalk between pancreatic cancer cells and dendritic cells

Exosomes are nanovesicles of endocytic origin that are released by many cell types into the extracellular microenvironment. Exosomes contain proteins, mRNAs, microRNAs and lipids and play a central role in intercellular communication [23]. Pancreatic cancer cells and DCs release exosomes that influence the tumor-immune function. In a mathematical model, it was reported that pancreatic cancer cells shed exosomes that inhibit immune responses by DCs, while DCs secrete exosomes that induce apoptosis of pancreatic cancer cells [24]. A previous study revealed that pancreatic cancer-derived exosomes attenuate DC-mediated tumor suppressive responses initiated by TLR4 [25]. Furthermore, pancreatic cancer-derived exosomes have been shown to inhibit regulatory factor X-associated protein expression through miR-212-3p. This may result in decreased expression of major histocompatibility complex (MHC) II and lead to immune tolerance of DCs [26]. Thus, tumor-derived exosomes regulate host immunosuppression by influencing the function and differentiation of DCs [27].

### Prognostic role of dendritic cells

The numbers of circulating DCs have been found to be an independent prognostic factor for prolonged survival in pancreatic cancer [12, 13]. However, distinct subsets of DCs may have diverging prognostic potential. For example, an increased level of immune tolerant immature DCs were shown to result in shorter survival [28].

### Treatment with dendritic cell-based vaccines

**Fig. 1:**
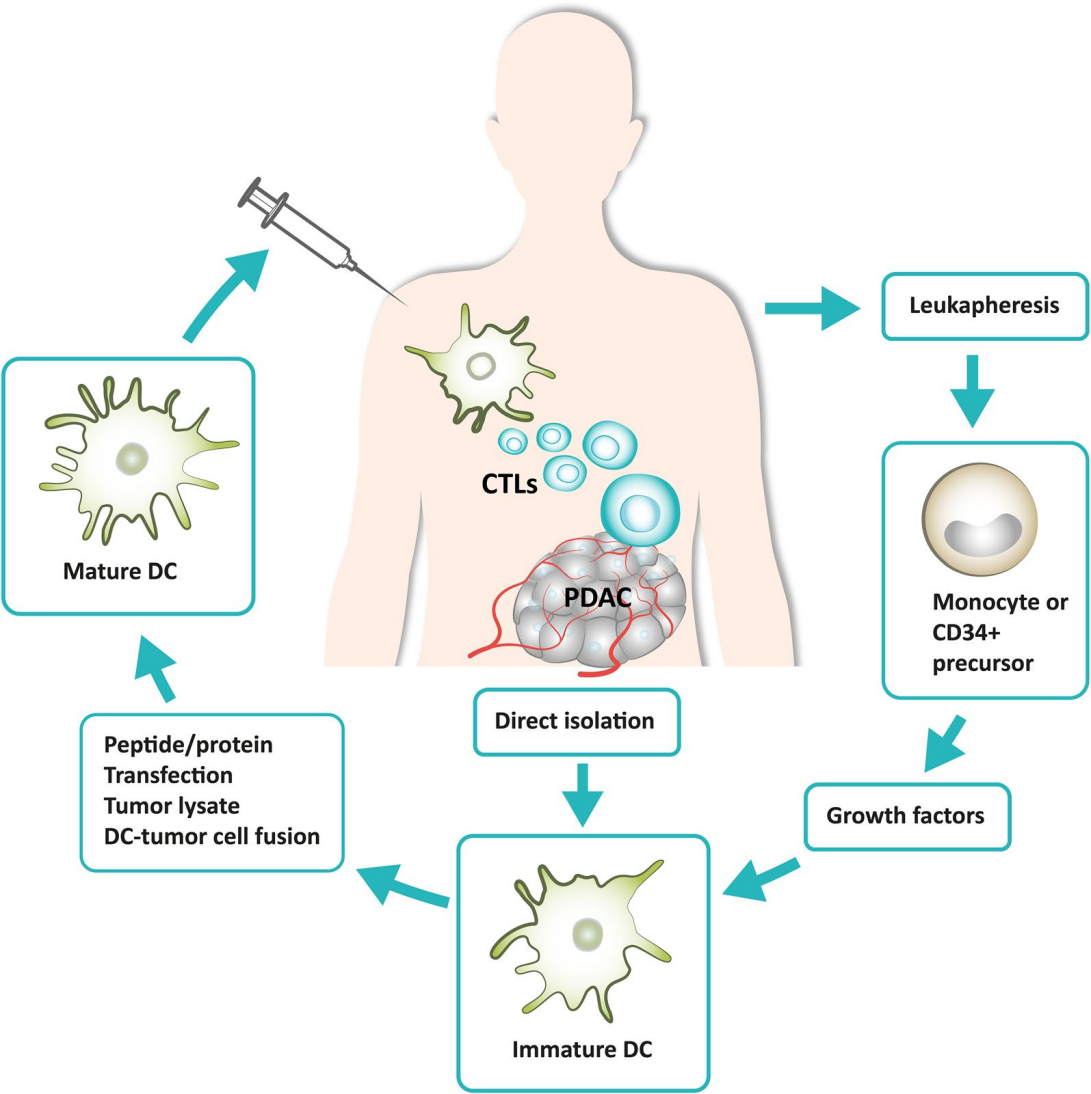
Principles of dendritic cell-based immunotherapy in pancreatic cancer, including extraction, priming and re-injection of dendritic cells. *CTL* cytotoxic T lymphocyte, *DC*, dendritic cell, *PDAC* pancreatic ductal adenocarcinoma

The basic idea of tumor vaccination is to confront (pulse) antigen presenting cells such as DCs with tumor-associated antigens to achieve presentation and thereby induce cytotoxic T cells. Different methods exist to pulse DCs: (a) synthetic peptides or purified proteins, (b) DNA, RNA or viruses (transfection), (c) tumor lysates or autophagosomes or (d) fusing tumor cells with DC (Fig. 1).

The use of peptides, DNA or RNA as a pulsation media results in a highly specific immune response. However, the applicable antigens need to be present in the patient, which reduces the range of treatable cases and requires genetic/proteomic analyses. Even so, a risk of cross-reactivity remains. Furthermore, peptides are restricted to a specific antigen presenting HLA molecule, which in turn narrows the number of benefiting patients. Genomic media allows for a broader selection of presenting HLA molecules and might therefore benefit a wider range of patients. However, (c)DNA requires entry into the nucleus and RNA is unstable, which are known limitations [29].

Tumor lysates allow for a broad selection of tumor associated antigens (TAAs) and thereby a more comprehensive tumor attack. However, the exact mechanisms and target structures of this method often remain unclear, and the risk of cross reactivity might be high.

Fusion of tumor cells with DCs is an interesting method to improve tumor antigen presentation. The approach also results in a wide range of presented antigens, but clinical studies are necessary to assess efficacy.

### Tumor associated antigens in pancreatic cancer

To date, different TAAs in pancreatic cancer have been discovered and are currently under investigation. Despite mostly having been investigated in vitro or in animal models, the first clinical data are already present (Table 1).

**Table 1:** Published series on dendritic cell-based immunotherapy in pancreatic cancer

Tumor-associated antigen	Year	N^a^	Stage	Pulsation	R	SD	PD	Median survival (months)
MUC1								
Pecher et al. [31]	2002	2	Unresectable	cDNA		0	2	
Kondo et al. [32]	2008	20	Unresectable	Peptide	1	5	14	9.8^b^
Lepisto et al. [33]	2008	12	Resectable	Peptide	4			26
Rong et al. [34]	2012	7	Unresectable	Peptide		0	7	
Shindo et al. [35]	2014	42	Unresectable	mRNA	4	22	16	13.9
WT1								
Koido et al. [37]	2014	10	Unresectable	Peptide		7	3	
Takakura et al. [39], Tsukinaga et al. [40]	2015	7	Unresectable	Peptide		6	1	10.8
Mayanagi et al. [38]	2015	10	Unresectable	Peptide		6	4	8.3
Okamoto et al. [41]	2016	255	Unresectable	Peptide				9.9
Mesothelin								
Thomas et al. [43]	2004	14	Unresectable	Peptide	Immunological response in 3 patients			
KRAS								
Gjertsen et al. [45]	1996	5	Unresectable	Peptide		3	2	5
hTERT, CEA, survivin								
Mehrotra et al. [55]	2017	12	Unresectable	Peptide				7.7
CEA, HER2, WT1								
Kimura et al. [56]	2012	49	Unresectable	Peptide	7	10	32	11.8
CEA, MUC1								
Nakamura et al. [59]	2009	12	Unresectable	Peptide, lysate				9
Tumor cell lysate								
Bauer et al. [61]	2011	12	Unresectable	Lysate	1	2	9	10.5

*PD* progressive disease, *R* partial or complete response to therapy, *SD* stable disease

^a^Only patients with pancreatic cancer were considered.

^b^Mean survival time

#### MUC1

MUC1 is a glycoprotein that is expressed in nearly all pancreatic cancers [30]. Transfected DCs with liposomal MUC1 cDNA were evaluated as a vaccine in 10 patients with metastatic breast, pancreatic and ampullary cancer [31]. The study showed the feasibility and toleration of tumor vaccination and reported an increased cytotoxic T-lymphocyte (CTL) response in the pancreatic cancer patients. The clinical benefit of MUC1-DCs and MUC1-CTLs was evaluated in 20 patients with unresectable or recurrent pancreatic cancer. Complete response was noted in 1 patient, and stable disease noted in 5 patients. The mean survival was 9.8 months [32]. Another study evaluated the clinical efficacy of a MUC1 peptide-loaded DC vaccine in 12 pancreatic and biliary cancer patients following surgical resection. The vaccine was well tolerated and no toxicity was observed. Four vaccinated patients are alive up to 5 years after surgery [33]. MUC1-peptide-pulsed DCs were evaluated in the treatment of 7 patients with metastatic or recurrent pancreatic cancer. The vaccination was well tolerated and capable of inducing immunological response, but no clinical benefit was noted [34]. Combination therapy using MUC1-mRNA-transfected DCs, MUC1-CTLs and gemcitabine was evaluated in 42 patients with unresectable or recurrent pancreatic cancer. Mean survival was 13.9 months. Patients who received high dose MUC1-DCs and MUC1-CTLs had significantly enhanced survival (16.5 months vs 5.7 months; p < 0.001) [35]. These data suggest that MUC1 is a promising TAA, considering both the reported feasibility and to some extend the effectiveness of DC-based MUC-1 specific immunotherapy.

#### WT1

Wilms tumor gene (WT1) encodes a zinc finger transcription factor that plays an important role in cell growth and differentiation. The WT1 protein is highly expressed (75%) in pancreatic cancers [36]. Treatment with DCs pulsed with WT1 peptides and chemotherapy in advanced pancreatic cancer has been evaluated in several small series from Japan [37–40]. The protocol was subsequently presented as a retrospective, multicenter analysis including 255 patients [41]. The median overall survival from diagnosis was 16.5 months and 9.9 months following treatment with standard chemotherapy combined with peptide-pulsed DC vaccines. The median survival time of the patients with positive delayed type hypersensitivity skin reaction was significantly prolonged compared to that of the patients with negative reaction (p = 0.015). These findings based on WT1-specific immunotherapy are promising, but more research is needed to stratify patient and determine responders.

#### Mesothelin

Mesothelin has been found to be overexpressed in the majority of pancreatic tumors, with a detection rate ranging from 60 to 100% [30, 42]. A vaccine with allogeneic GM-CSF-secreting pancreatic tumor cell lines was found to induce delayed type hypersensitivity responses in 3 out of 14 treated pancreatic cancer patients [43]. The vaccine was found to recruit DCs to the site of vaccination and stimulate CD8+ T cells by a cross-priming mechanism involving mesothelin.

#### KRAS

KRAS is mutated in more than 90% of pancreatic cancer and is a central driver of pancreatic tumor growth and progression [44]. Synthetic RAS peptide has been used as a vaccine in 5 patients with advanced pancreatic carcinoma. An immune response against the immunizing RAS peptide could be induced in 2 patients [45]. The median survival was 10.5 months for the 2 responding patients, compared to 4.5 months for the non-responding patients. Most KRAS mutations in pancreatic cancer are located in codon 12, while mutations in codon 13 and 61 are much less common [46]. Development of new vaccine protocols that target the individual patient’s specific RAS mutation may further improve immunogenicity and ultimately prove clinically beneficial.

#### Other antigens

Other peptides and proteins have been used to pulse DCs in order to induce a tumor-specific immune response. CA 19-9 is a classic tumor marker, present in approximately 80% of pancreatic cancer patients [47]. It has been found that DCs pulsed with the CA 19-9 protein or CA 19-9 containing serum have the ability to induce cytotoxic activity against pancreatic cancer cells in vitro [48, 49]. Further preclinical data support the immunogenic potential of Trop2 [50], MUC-4 [51], pathological bile salt-dependent lipase (pBSDL) [52, 53] and α-Enolase [54]. In patients, human telomerase reverse transcriptase (hTERT) [55], carcinoembryonic antigen (CEA) [55–57], survivin [55], HER2 [56, 57], CA-125 [56] and α-fetoprotein [57] have also been tested in small series. Most of these antigens have been tested in a combinatorial approach using multiple peptides as targets for DC-based vaccination. This is a promising approach, which addresses the problem of impaired range of targets in peptide-, DNA- or RNA-based vaccination protocols. However, there is also need to compare the efficacy of individual peptides.

#### Tumor cell lysate

In addition to the use of peptides as pulsation substance, lysed tumor cells can be used. Tumor lysate can be generated by multiple freeze–thaw cycles or UV-irradiation of tumor cells. The potential benefits include a broader selection of TAAs and thereby a more comprehensive tumor attack, but the exact mechanisms and target structures of this method often remain unclear. Also, it cannot be excluded that non-tumor-specific antigens are expressed by DCs within this method, resulting in cross- or auto-reactive CTLs [58]. Nonetheless, whole tumor-lysate for pulsing DCs has been shown to be feasible, safe and potentially beneficial as a vaccination-method in pancreatic cancer [59–62]. This strategy provides a reproducible pool of most tumor antigens suitable for patient use, independent of MHC haplotypes or autologous tumor tissue availability. However, optimizing autologous tumor cell lysate preparation is crucial in order to enhance efficacy.

#### Cellular fusion

Fusion of DCs with pancreatic tumor cells potentially acts synergistically to improve antigenicity and antigen presentation in order to induce tumor-specific cytotoxic immunity. So far, most data using these cellular hybrids have been reported in experimental settings of pancreatic cancer [63–65].

## Conclusion

Pancreatic cancer is associated with an immune tolerant state, which is mediated by a complex shift in the number, phenotype and function of multiple immune cells, including DCs. Subpopulations of DCs are altered in pancreatic cancer related to effects by the tumor cells as well as the microenvironment. Pulsation of DCs with a wide range of antigens has been shown to be effective in initiating adaptive, antigen-specific immune responses, but there remain many unresolved questions. For example, more research needs to be conducted into the optimal sequence and interval of vaccination, the role of immune adjuncts and the potential synergies with conventional treatment such as surgery, chemotherapy and radiation. In the future, the use of novel vaccine protocols based on the individual patient’s tumor phenotype may lead to long-term clinical benefit.

## Abbreviations

DC: dendritic cell
HLA: human leucocyte antigen
MDSC: myeloid derived suppressor cell
MHC: major histocompatibility complex
MUC1: mucin 1
TAA: tumor associated antigen
TAP: transporter for antigen presentation
Treg: regulatory T cell
WT1: Wilms tumor 1
